# Fucosylation of LAMP-1 and LAMP-2 by FUT1 correlates with lysosomal positioning and autophagic flux of breast cancer cells

**DOI:** 10.1038/cddis.2016.243

**Published:** 2016-08-25

**Authors:** Keng-Poo Tan, Ming-Yi Ho, Huan-Chieh Cho, John Yu, Jung-Tung Hung, Alice Lin-Tsing Yu

**Affiliations:** 1 Institute of Microbiology and Immunology, National Yang-Ming University, Taipei, Taiwan; 2 Institute of Stem Cell and Translational Cancer Research, Chang Gung Memorial Hospital at Linkou, Taoyuan, Taiwan; 3 Chang Gung University, Taoyuan, Taiwan; 4 Department of Pediatrics, University of California in San Diego, San Diego, CA, USA

## Abstract

Alpha1,2-fucosyltransferases, FUT1 and FUT2, which transfer fucoses onto the terminal galactose of *N*-acetyl-lactosamine via *α*1,2-linkage have been shown to be highly expressed in various types of cancers. A few studies have shown the involvement of FUT1 substrates in tumor cell proliferation and migration. Lysosome-associated membrane protein 1, LAMP-1, has been reported to carry alpha1,2-fucosylated Lewis Y (LeY) antigens in breast cancer cells, however, the biological functions of LeY on LAMP-1 remain largely unknown. Whether or not its family member, LAMP-2, displays similar modifications and functions as LAMP-1 has not yet been addressed. In this study, we have presented evidence supporting that both LAMP-1 and 2 are substrates for FUT1, but not FUT2. We have also demonstrated the presence of H2 and LeY antigens on LAMP-1 by a targeted nanoLC-MS^3^ and the decreased levels of fucosylation on LAMP-2 by MALDI-TOF analysis upon FUT1 knockdown. In addition, we found that the expression of LeY was substantial in less invasive ER+/PR+/HER− breast cancer cells (MCF-7 and T47D) but negligible in highly invasive triple-negative MDA-MB-231 cells, of which LeY levels were correlated with the levels of LeY carried by LAMP-1 and 2. Intriguingly, we also observed a striking change in the subcellular localization of lysosomes upon FUT1 knockdown from peripheral distribution of LAMP-1 and 2 to a preferential perinuclear accumulation. Besides that, knockdown of FUT1 led to an increased rate of autophagic flux along with diminished activity of mammalian target of rapamycin complex 1 (mTORC1) and enhanced autophagosome–lysosome fusion. This may be associated with the predominantly perinuclear distribution of lysosomes mediated by FUT1 knockdown as lysosomal positioning has been reported to regulate mTOR activity and autophagy. Taken together, our results suggest that downregulation of FUT1, which leads to the perinuclear localization of LAMP-1 and 2, is correlated with increased rate of autophagic flux by decreasing mTOR signaling and increasing autolysosome formation.

Alpha1,2-fucosyltransferases (FUT1 and FUT2) are key enzymes to catalyze the transfer of fucose onto the terminal galactose of type 1 or type 2 disaccharide (Gal (*β*1,3 or 1,4) GlcNAc-R) via *α*1,2-linkage,^1, 2^ resulting in H type 1 (H1) or H type 2 (H2) antigens. In general, FUT1 preferentially generates H type 2 antigens, whereas FUT2 shows preference for H type 1.^3^ The H antigens can be further substituted by adding a fucose onto the *N*-acetylglucosamine of type 1 or type 2 precursors in *α*1,4- or *α*1,3-linkage, giving rise to Lewis B (LeB) and Lewis Y (LeY) antigens, respectively.^4^ The aberrant *α*1,2-fucosylation is a hallmark of multiple types of cancers. Several reports have demonstrated that overexpression of FUT1 is associated with enhanced tumor cell proliferation and tumorigenicity, whereas silencing of FUT1 exerts the opposite effects.^5, 6, 7, 8^ In addition, the involvement of FUT1 in other cellular processes such as adhesion,^4, 9^ migration,^10^ apoptosis^1^ and angiogenesis have also been demonstrated.^11, 12^ Although the importance of *α*1,2-fucosytransferase in cancer biology processes has been reported, the molecular mechanisms involved remains largely unclear. Thus, it is worthwhile to identify the target proteins of *α*1,2-fucosytransferase and to gain insight into their biological functions.

Lysosome-associated membrane protein 1 (LAMP-1) and its family member LAMP-2 share 37% amino-acid sequence homology and both are highly glycosylated type I transmembrane proteins with 18 and 16 potential *N*-linked glycosylation sites, respectively. Both LAMP-1 and 2 are mainly found in the lysosomes and late endosomes,^13^ and are known to maintain the lysosomal acidification and lysosomal membrane integrity.^13, 14^ In addition to their role in lysosomal biogenesis, it has also been reported that both LAMP-1 and 2 or LAMP-2 alone is associated with autophagosome accumulation and biogenesis.^15, 16, 17^ The presence of extensive *N*-glycosylation on both LAMP-1 and 2 has been suggested to protect them from the lysosomal proteolysis.^18, 19^ On the other hand, greater levels of poly-*N*-acetyllactosamines^20, 21^ on LAMP-1 and 2 are correlated with decreased cellular differentiation^22, 23, 24^ and enhanced metastatic potential.^25, 26^ It has been suggested that the terminal glycan residue, sLeX, on LAMP-1 and 2 is involved in cellular adhesion, tumor invasion and metastasis by binding to the E-selectin expressing endothelial cells,^25, 27^ whereas LeY termini on LAMP-1 are believed to be associated with breast cancer cell migration.^28^ Although the expression of LeY termini on surface LAMP-1 in breast cancer cells have been reported to be 30-fold higher than that of non-tumorigenic mammary epithelial cells,^28^ the presence of LeY on LAMP-2, and the role of LeY in the biological functions of LAMP-1 and 2 remain to be delineated.

In this study, we have demonstrated that LAMP-1 and 2 are target proteins for FUT1 but not FUT2. We have also demonstrated that LeY antigens carried by LAMP-1 and 2 in weakly invasive breast cancer cells are significantly more abundant than those from highly invasive cancer cells. Notably, we found that a more prominent perinuclear accumulation of LAMP-1/2(+) vesicles in FUT1 knockdown cells as compared with control cells with peripheral distribution. More importantly, our results reveal that FUT1 knockdown, which leads to increased perinuclear positioning of lysosomes, is accompanied by an increased autophagosome–lysosome fusion and a decreased mTORC1 activity that may, in turn, be associated with an increased rate of autophagic flux.

## Result

### Both LAMP-1 and 2 are substrates of FUT1

**Figure 1:**
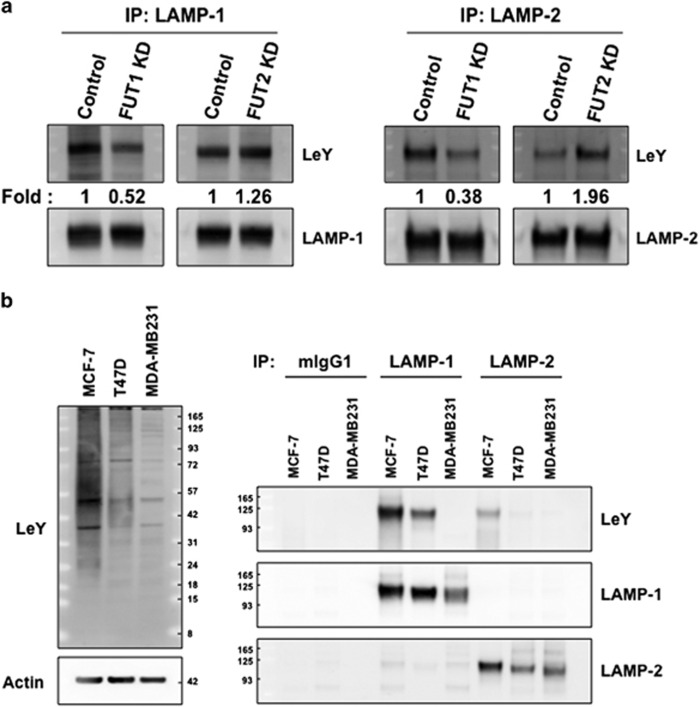
LAMP-1 and 2 are substrates of FUT1. (**a**) Expression levels of LeY antigens on immunoprecipitated LAMP-1 and 2 from control, FUT1 and FUT2 knockdown cells. Both LAMP-1 and 2 were immunoprecipitated from FUT1 and FUT2 silenced T47D cells and then immunobloted with antibodies against LeY. The same blots were stripped and reprobed for LAMP-1 or LAMP-2. The ratios of LeY to immunoprecipitated LAMP-1 or LAMP-2 were quantified and fold changes relative to control were shown as indicated. (**b**) Immunoblot analysis of LeY levels in MCF-7, T47D and highly metastatic MDA-MB-231 cells (left panel). Actin was used as loading control. Expression levels of LeY antigens on immunoprecipitated LAMP-1 and 2 from MCF-7, T47D and MDA-MB-231 cells (right panel). LAMP-1 and 2 were immunoprecipitated from the indicated cells, and immunobloted with anti-LeY, anti-LAMP-1 or anti-LAMP-2 antibodies used in (**a**)

It is known that both LAMP-1 and 2 contain numerous putative *N*-glycosylation sites and five (N shown in red in Supplementary Figure S1a) of these sites are conserved between LAMP-1 and 2. All of these *N*-glycosylation sites are exclusively located in their luminal ectodomain. To examine whether LAMP-1 is a target of *α*(1,2)-fucosytransferases, FUT1 and FUT2 were transiently overexpressed in breast cancer cell line T47D breast cancer cells in which the mRNA expression of FUT1 and FUT2 was about ~8401 and ~5490-fold of the control groups, respectively (Supplementary Figure S2a). The transfected cells were then treated with alkynyl fucose for 3 days followed by immunoprecipitation of LAMP-1 for ‘On-Membrane Click Reaction.' As shown in Supplementary Figure S1b, the fucosylation of LAMP-1 with different linkage types was detected in T47D cells overexpressing either FUT1, FUT2 or control vector. Notably, the extent of fucosylation on LAMP-1 in cells overexpressing FUT1 was about 1.8-fold greater than those of the control, whereas the extent of fucosylation on LAMP-1 in cells overexpressing FUT2 showed no significant difference. This suggests that LAMP-1 may be a target protein for FUT1, but not FUT2. In addition, T47D cells, which express both FUT1 and FUT2 activities, were treated with siRNA for FUT1 or FUT2 to silence FUT1 or FUT2 expression, to 12.6% and 5.6%, respectively, of negative controls (Supplementary Figures S2b and S1c). As expected, a significant decrease of LeY levels on both LAMP-1 and 2 was only found in FUT1 knockdown cells, but not FUT2 knockdown cells, when compared with the control cells (Figure 1a). This result further confirms that both LAMP-1 and 2 can be modified by FUT1 but not FUT2. Moreover, we also found that the expression of LeY in less invasive MCF-7 and T47D cells (ER and PR positive) was more abundant than that in the invasive triple-negative MDA-MB-231 cells, and that the LeY levels in these cells are closely correlated to the levels of LeY carried by LAMP-1 and 2 (Figure 1b). Collectively, these results suggest that FUT1, but not FUT2, is the main catalyst for both LAMP-1 and 2, which regulates the levels of LeY carried by LAMPs.

### Mass spectrometric analysis shows a decrease in terminal fucosylation on both LAMP-1 and 2 upon FUT1 knockdown

**Figure 2:**
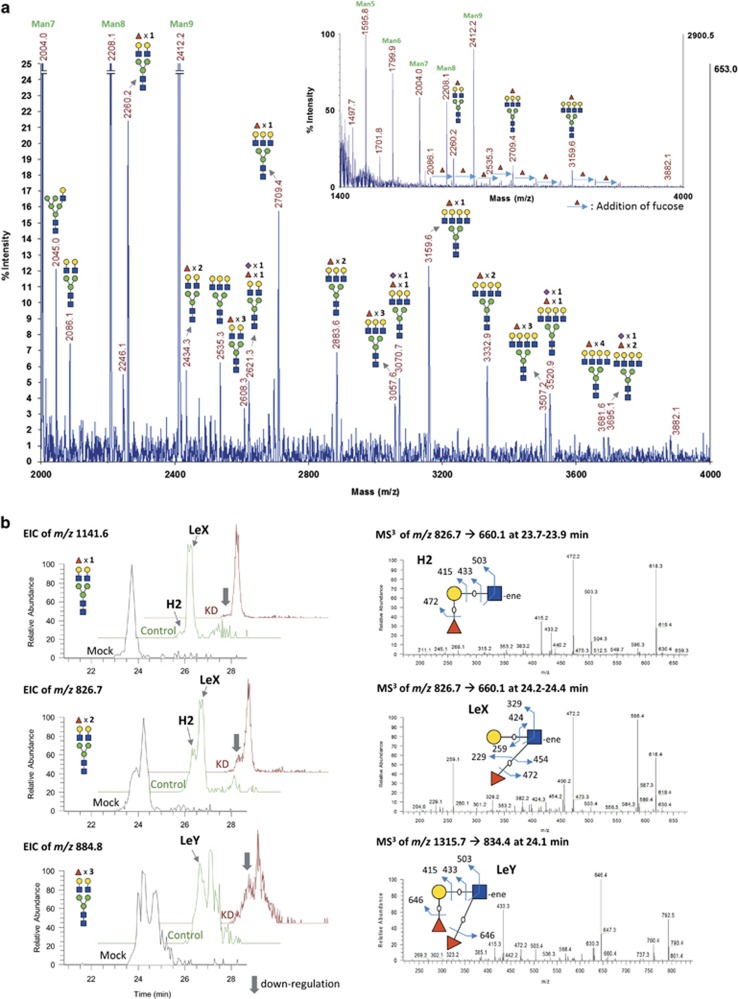
Characterization of *N-*glycans on purified LAMP-1. (**a**) MALDI-TOF mass spectrum of permethylated *N-*glycans of LAMP-1 expressed in mock-treated T47D cells. The insert graph showed full range MS spectrum with predominant high-mannose and minor complex-type *N-*glycans on LAMP-1 glycoproteins. The bi-, tri- and tetra-antennary complex *N-*glycans of LAMP-1 were highly fucosylated with up to four fucose residues attached. The colored symbol and nomenclature for glycan structure follow the designation of Consortium for Functional Glycomics (http://www.functionalglycomics.org/static/consortium/Nomenclature.shtml). (**b**) LC-MS^3^ analysis of bi-antennary *N-*glycans of LAMP-1 from control and FUT1 knockdown T47D cells. The EICs in the left panel showed the sodiated molecular ions of bi-antennary *N-*glycans carried with one (*m/z* 1141.6, [M+2Na]^2+^), two (*m/z* 826.7, [M+3Na]^3+^) or three (*m/z* 884.8, [M+3Na]^3+^) fucose residues of purified LAMP-1 from mock, control or FUT1 knockdown cells. The EICs were reconstructed by ion intensities within 20 p.p.m. accuracy of theoretical mass value. The major fragment ions of H type 2 (H2), Lewis X (LeX) and LeY glycotopes are schematically illustrated on the right panel. Notably, those bi-antennary *N-*glycans with distinct terminal glycotopes were eluted at different retention times (RTs). The fragmentation was annotated according to the Domon and Costello nomenclature^54^

**Figure 3:**
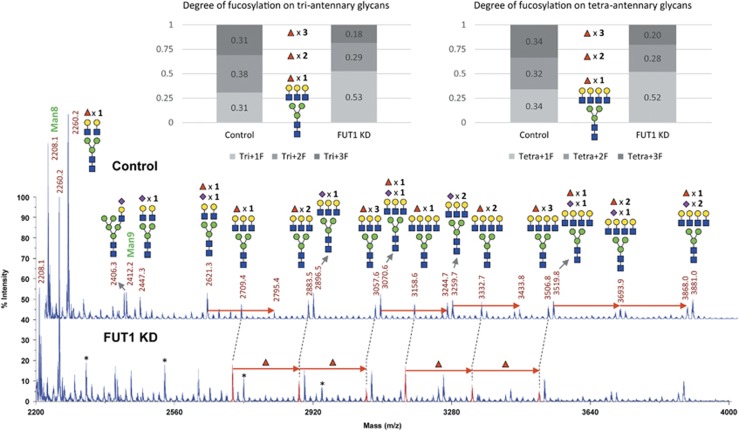
MALDI-TOF mass spectra of complex *N-*glycan from purified LAMP-2 expressed in control and FUT1 knockdown T47D cells. The changes of fucosylation level on tri- and tetra-antennary *N-*glycans were illustrated as relative abundance of each glycoform (as shown in different shades of gray boxes). Notably, the levels of fucosylation decreased in both tri- and tetra-antennary *N-*glycans of LAMP-2 in FUT1 knockdown cells as compared with that in control cells. The molecular ions representing complex glycans with degree of fucosylation were highlighted in the lower spectrum as red peaks at *m/z* 2709, 2883 and 3057 for tri-antennary and *m/z* 3158, 3332 and 3506 for tetra-antennary N-glycans, respectively. The relative ratio of each glycoform is given in percentage of total sum of peak intensities of tri- and tetra-antennary glycans in the MS spectra. The colored symbol and nomenclature for glycan structure are based on the designation of Consortium for Functional Glycomics as described in Figure 2a. Peaks labeled with asterisks represent polyhexose ladder contaminations that were negligible for overall analysis

To analyze the changes in *N-*glycan profiles of LAMP-1 and 2 upon FUT1 knockdown, the endogenous LAMPs from T47D cells were immunoprecipitated and separated by SDS-PAGE. The *N-*glycans were then enzymatically released by in*-*gel PNGase F treatment with subsequent reduction and permethylation for MALDI-TOF MS and nanoLC-MS/MS analysis. In MALDI-TOF profiles, the major *N-*glycosylation structures found on LAMP-1 were high-mannose (predominant) and complex-type (minor) *N-*glycans (Figure 2a); whereas LAMP-2 showed predominantly complex bisecting-type *N-*glycans (Figure 3; ions at *m/z* 2260). As shown in Figure 2a, the complex-type *N-*glycans of LAMP-1 with either bi-, tri- or tetra-antennary structures exhibited high levels of fucosylation (with up to four fucose residues) along with relatively low levels of sialylation. However, fucosylation on LAMP-2 with 2 to 3 fucoses were only found on the tri- and tetra-antennary *N-*glycans. Although the complex type *N-*glycans on LAMP-2 were relatively low as compared with those of LAMP-1, a significant decrease in the extents of fucosylation on LAMP-2 was still observed upon FUT1 knockdown (Figure 3). To characterize the terminal fucosylated epitopes modified by FUT1, we further used a targeted nanoLC-MS^3^ method to enable specific linkage analysis and quantitation of the terminal fucosylated glycotopes of *N-*glycans on LAMP-1. In this analysis, the complex *N-*glycans of LAMP-1 were limited to bi-antennary structures and to those with 1 to 3 fucose residues that were preset as precursor ion candidates for data-dependent MS/MS acquisition. Among the MS/MS product ions afforded by fucosylated precursors, B-fragment ion at *m/z* 660.3 was further selected for MS^3^ analysis to characterize the terminal glycotopes. As shown in Figure 2b, the extracted ion chromatogram (EIC) of *m/z* 826.7 for bi-antennary structures with additional two fucose residues showed peak splitting at retention time 23.8 and 24.3 min. The earlier peak was further identified as bi-antennary structures with H type 2 glycotopes by MS^3^ spectrum of fragment ions at *m/z* 415, 433 and cross-ring fragment ions at *m/z* 503 (^3,5^A), whereas the later peak was determined as Lewis X (LeX) glycotopes according to the diagnostic ions at *m/z* 259, 454 and 329 (^3,5^A). Comparison of the EICs for the representative bi-antennary glycans of LAMP-1 among mock, control or FUT1 knockdown cells revealed a significant difference in the intensities of these peaks. Thus, our data clearly demonstrated that the expression of H2 glycotopes on LAMP-1 was reduced in FUT1 knockdown cells as compared with those of the mock or control cells. Similar to the tandem mass analysis of H2 and LeX structural isomer, the B-fragment ions (*m/z* 834.4) in MS/MS spectrum, which could only be found in bi-antennary glycans with three fucose residues, was sijmilarly selected at the retention time 24.1 min for MS^3^ analysis of LeY glycotopes. The identification of LeY was mainly based on diagnostic fragment ions at *m/z* 415, 433, 646 and cross-ring fragment ions at *m/z* 503 (^3,5^A) in the MS^3^ spectrum. Similar to H2 glycotopes, EIC of *m/z* 884.8 for the representative glycans of LAMP-1 showed a decrease in the intensity of LeY glycotopes upon FUT1 knockdown. This is consistent with our result in Figure 1a that the expression of LeY on LAMP-1 was reduced upon FUT1 knockdown. Taken together, these MS results further verified that FUT1 is responsible for the terminal fucosylation of H2 and LeY found on both LAMP-1 and 2. Supplementary Table S1 summarizes the results of fucosylation changes in LAMP-1 or LAMP-2 upon FUT1 knockdown detected by various analytical methods.

### Downregulation of FUT1 leads to accumulation of LAMP-1/2(+) vesicles at perinuclear area

**Figure 4:**
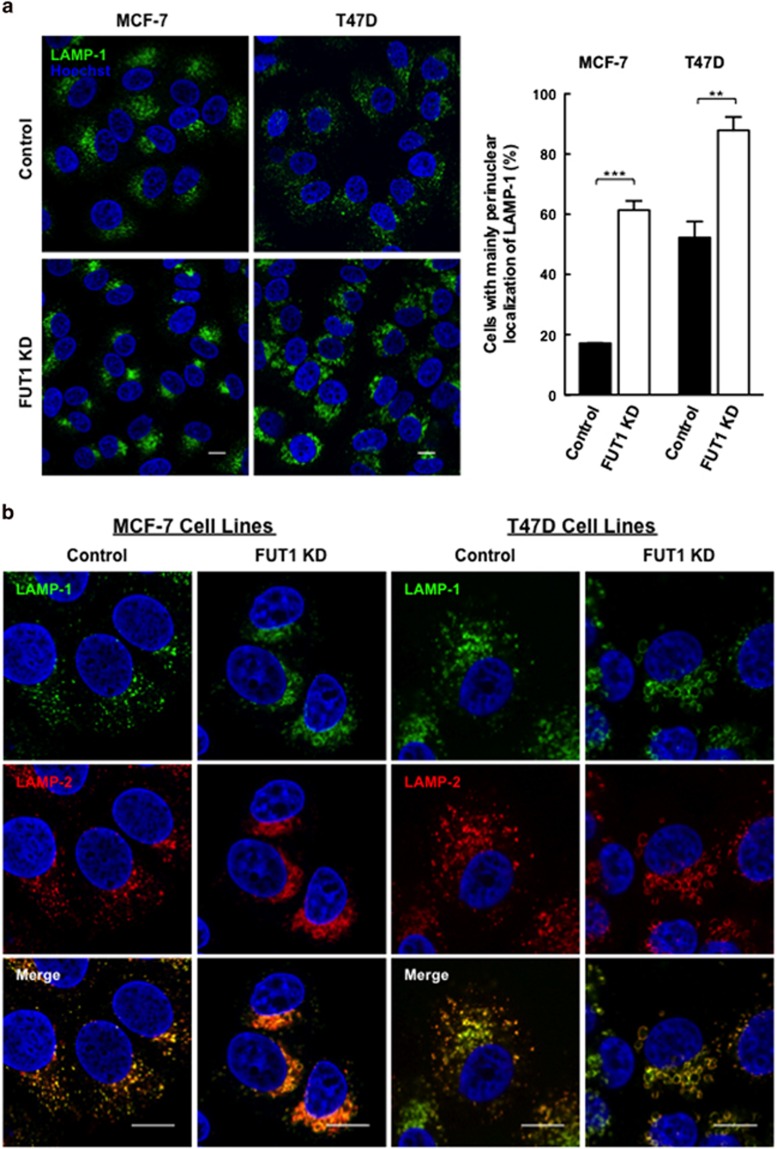
Downregulation of FUT1 leads to accumulation of LAMP-1/2(+) vesicles at perinulear area of MCF-7 and T47D cells. (**a**) Immunofluorescence staining for LAMP-1 in control and FUT1 knockdown cells with anti-LAMP-1 (green) and Hoechst (nucleus, blue). Stained cells were then analyzed by confocal microscopy at × 63 magnification (MCF-7 cells) and × 100 magnification (T47D cells). Scale bars, 10 *μ*m. Histogram shows the percentage of control (black bars) and FUT1 knockdown (white bars) cells with predominantly perinuclear localization of LAMP-1. Data shown are the mean±S.E.M. of three independent experiments (***P<0.001; **P<0.01). (**b**) Colocalization of LAMP-1 and 2 in perinuclear area of FUT1 knockdown MCF-7 and T47D cells. Control and FUT1 silenced T47D/MCF-7 cells were co-stained with anti-LAMP-1 (green) and anti-LAMP-2 (red), and nuclei were stained with Hoechst (blue). Colocalization was visualized by confocal microscopy at × 63 magnification (MCF-7 cells) and × 100 magnification (T47D cells) with 3X optical zoom. Representative colocalization signals were shown in the merged image as yellow. Scale bars, 10 *μ*m

Upon silencing of FUT1 in MCF-7 and T47D breast cancer cells, we observed a striking change in the subcellular distribution patterns of LAMP-1 and 2 by immunofluorescence staining. As shown in Figure 4a, LAMP-1 staining in the control cells appeared as vesicle-like structures and distributed randomly in the cytoplasm. In contrast, LAMP-1(+) vesicles in FUT1 knockdown cells mostly accumulated in the perinuclear region. Quantitative analysis showed that the proportion of cells with predominantly perinuclear LAMP-1(+) vesicles increased from 17.16±0.1% and 52.27±5.3% in control MCF-7 and T47D cells, respectively, to 61.39±3% and 87.93±4.4% in FUT1 silenced MCF-7 and T47D cells (*P*=0.0001, *P*=0.0068), respectively. As expected, the perinuclear-dominant distribution of LAMP-1 upon FUT1 knockdown was also observed in LAMP-2(+) vesicles, which colocalized with LAMP-1, as seen in the control cells (Figure 4b). In addition, LAMP-1/2(+) vesicles in FUT1 knockdown cells tended to cluster together leading to larger lysosomal vesicles as compared with control. Here, we also stained the T47D cells with a lysosomotropic dye acridine orange to characterize the changes in the distribution of LAMP-1/2(+) vesicles. As shown in Supplementary Figure S3, those vesicle structures observed in both control and FUT1 knockdown cells exhibited characteristics of acidic lysosomal compartments with orange red signals. Thus, the altered distributions of LAMP-1 and 2 upon FUT1 knockdown could reflect the changes of lysosomal positioning. Taken together, these results suggest that FUT1 silencing alters both the morphology and positioning of lysosomes with relocalization of the enlarged LAMP-1/2(+) vesicles to the perinuclear region.

### Downregulation of FUT1 is correlated with an increased rate of autophagic flux

**Figure 5:**
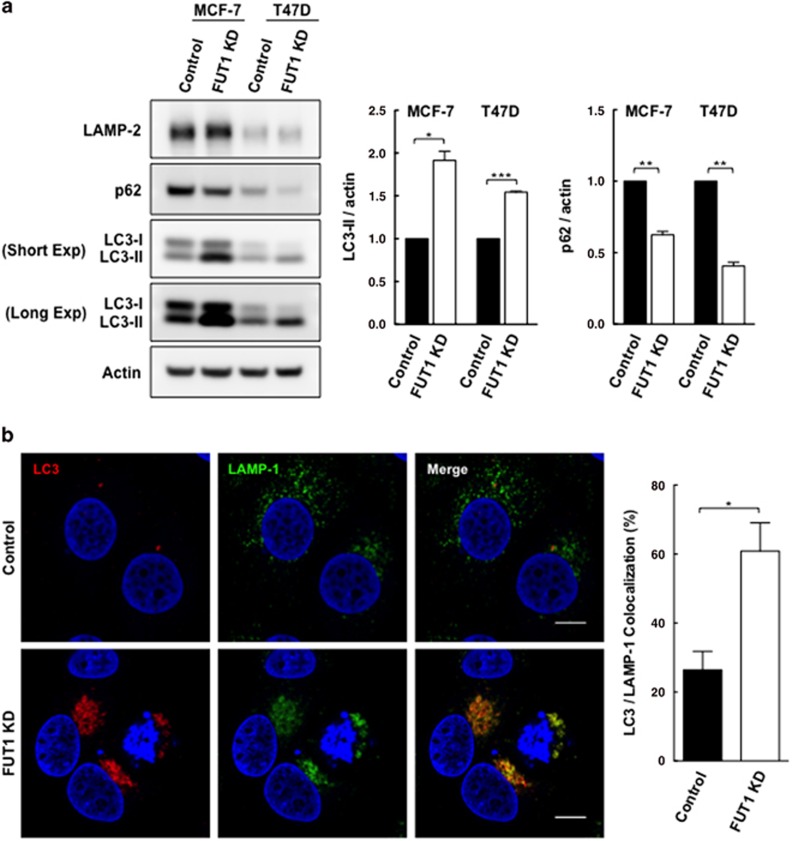
Knockdown of FUT1 is associated with an increase in autophagic flux. (**a**) Immunoblot analysis of LC3-II and p62 levels in control and FUT1 knockdown cells. Total cell lysates from MCF-7 and T47D cells transfected with control or FUT1 siRNAs were collected at 120 and 96 h post-transfection, respectively. Equal amounts of cell lysates were then loaded in each lane and separated by SDS-PAGE. Immunoblot analysis was performed with LC3 and p62 antibodies. Actin was used as a loading control. The intensity of LC3-II and p62 protein bands on immunoblot were quantified and normalized to actin, and the relative levels of protein expression were expressed as fold change by setting the control group value to 1. Values shown are mean±S.E.M. of three independent experiments (***P<0.001; **P<0.01; *P<0.05). (**b**) Downregulation of FUT1 enhanced the fusion of autophagosome and lysosomes in MCF-7 cells. Cells were co-stained with anti-LAMP-1 (green) and anti-LC3 (red) and nuclei stained with Hoechst (blue). Representative colocalization signals (referred to as autolysosomes) were shown in yellow in the merged. Magnification × 63, zoom: × 3. Scale bars, 10 *μ*m. Histogram shows the percentages of autolysosomes (LC3^+^/LAMP-1^+^) to autophagosomes (LC3^+^/LAMP-1^−^). Data are mean±S.E.M. of three independent experiments of >100 cells per group (*P<0.05)

**Figure 6:**
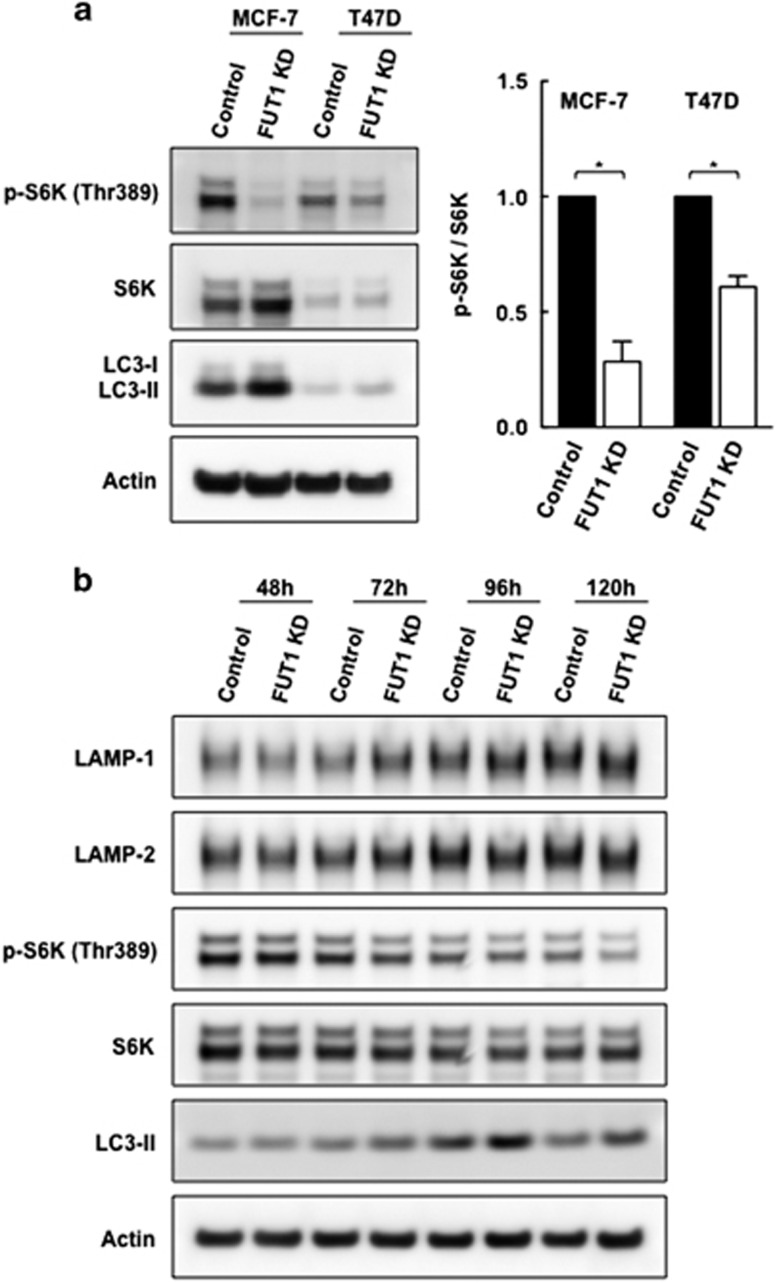
Knockdown of FUT1 is associated with decreased mTORC1 activity. (**a**) Representative immunoblots and quantitative analysis of mTOR activity in control and FUT1 knockdown cells. Lysates of MCF-7 and T47D cells transfected with control or FUT1 siRNAs were subjected to immunoblot analysis with antibodies against phospho-p70 S6K (Thr389) and total S6K. The resulting immunoblot bands were then quantified and computed as phospho-p70 S6K/total S6K ratio for mTOR activity. Values shown are mean fold change±S.E.M. of three independent experiments (*P<0.05). (**b**) Time course of mTORC1 activity in control and FUT1 knockdown MCF-7 cells. mTOR activity was determined by immunoblot analysis for the phosphorylation status of p70 S6K from 48 to 120 h after FUT1 knockdown. Levels of LAMP-1, LAMP-2 and LC3-II were analyzed in parallel over the indicated time points and actin was used as a loading control. Results are representative of two independent experiments

It has been reported that lysosomal positioning could regulate the initiation and termination stages of autophagic flux.^29, 30^ Thus, to further investigate the consequence of lysosomal positioning mediated by FUT1, we first examined the autophagic process in FUT1 knockdown cells. As shown in Figure 5, the levels of LC3-II increased to 1.92±0.11 (*P*=0.013) and 1.54±0.01-fold (*P*=0.0005) of control cells in FUT1 knockdown MCF-7 and T47D cells, respectively. However, the levels of autophagic substrate p62 decreased to 0.63±0.02 (*P*=0.004) and 0.41±0.03-fold (*P*=0.002) of control cells in FUT1 knockdown MCF-7 and T47D cells, respectively. On the other hand, we also measured the turnover of LC3-II in control and FUT1 knockdown cells with or without lysosomal inhibitor chloroquine (CQ). As shown in Supplementary Figure S4, autophagosome accumulation with CQ resulted in enhanced levels of LC3-II in both control and FUT1 knockdown cells but it was more pronounced in FUT1 knockdown cells. Taken together, our results suggest that FUT1 knockdown is probably associated with an increase in the rate of autophagic flux. To determine whether FUT1 exerts effect on autophagosome–lysosome fusion, we next examined the extent of colocalization of the endogenous LC3 (autophagosomal marker) with LAMP-1 (a representative of lysosomal compartment) in FUT1 knockdown cells. As shown in Figure 6b, the colocalization of LC3 puncta with LAMP-1 in control MCF-7 cells is only 26.4±5.4% as compared with approximately 60.9±8.2% colocalization in FUT1 knockdown cells (*P*=0.025), suggesting that knockdown of FUT1 could facilitate the fusion of autophagosomes and lysosomes to form degradative autolysosomes. It is noted that the LC3 puncta and LAMP-1 in FUT1 knockdown cells preferentially colocalized at perinuclear region. This further supports the previous notion that the emergence of perinuclear lysosomes may increase their accessibility for the autophagosomes to form autophagolysosomes.^30^

### Downregulation of FUT1 is associated with decreased mTORC1 activity

As lysosomal positioning has been reported to coordinate mTORC1 activity,^30, 31^ we thus examined whether knockdown of FUT1, which increased perinuclear lysosomes, was associated with a decrease in mTOR activity. As shown in Figure 6a, the phosphorylation status of p70 S6 kinase relative to the total S6K was reduced to 0.28±0.09 (*P*=0.015) and 0.6±0.05-fold (*P*=0.014) of the control cells in FUT1 knockdown MCF-7 and T47D cells, respectively, indicating suppression of mTOR activity upon FUT1 silencing. This was also confirmed with a time kinetic study of mTOR activity in which the activity of mTOR began to decline at 72 h after downregulation of FUT1 and continued to drop up to 120 h. Notably, the increase in LC3-II became evident at 72 h and continued to rise up to 120 h, whereas the levels of LAMP-1 and 2 remained unchanged throughout this time course (Figure 6b). To further define the correlation between mTOR activity and autophagy upon FUT1 knockdown, LC3-II levels in control and FUT1 knockdown cells were evaluated in the presence or absence of rapamycin alone or in combination with CQ. As shown in Supplementary Figure S4, autophagy activation with rapamycin was induced significantly in control cells with increased levels of LC3-II, whereas FUT1 knockdown had only modest effects. Upon treatment with both rapamycin and CQ, the levels of LC3-II in control and FUT1 knockdown cells were further elevated but more pronounced in FUT1 knockdown cells, suggesting the inhibitory effect of FUT1 on autophagic flux is partially attributed to mTOR activity. As the peripheral localization of lysosomes is reported to be associated with increased mTORC1 activity by bringing it to cell membrane Rheb activator,^30, 32, 33^ we thus further examined the distribution of mTOR in control and FUT1 knockdown cells. As shown in Supplementary Figure S5, mTOR together with LAMP-1 appeared to be accumulated at perinuclear region upon downregulation of FUT1, suggesting that the perinuclear positioning of mTORC1 may discourage its access to upstream signaling molecules, thereby decreasing its activity. Taken together, these results suggest that knockdown of FUT1 would facilitate the perinuclear accumulation of lysosomes, which is accompanied by diminished mTOR signaling and enhanced autophagic flux.

## Discussion

In this study, we combined the biochemistry, MALDI-TOF MS and nanoLC-MS^3^ analysis to characterize the alpha1,2-fucosylation on both LAMP-1 and 2 in breast cancer cells. Differing from the report of Jacques *et al.* that LAMP-1 is identified as a carrier for LeY antigens, our study has additionally demonstrated the presence of H2 and LeY antigens on LAMP-1 is mediated by FUT1 but not FUT2. Similarly, we have also identified the LAMP-1 family member, LAMP-2, as a novel substrate of FUT1 with LeY moiety attached. Topographically, we have discovered a striking change in the subcellular localization of LAMP-1 and 2 upon FUT1 knockdown in which LAMP-1 and 2 were preferentially accumulated at perinuclear region rather than being at the peripheral region, as seen in the control cells. On the other hand, we have found that knockdown of FUT1 results in an increased rate of autophagosome formation and degradation, which is accompanied by a decrease in mTORC1 (a known suppressor of autophagy) activity and an increase in autophagosome–lysosome fusion. As lysosomal positioning has been reported to coordinate mTOR activity and autophagy, the enhancement of autophagic flux in FUT1 knockdown cells appears to be the result of decreased mTOR signaling and increased autolysosome formation. Although LeY carried by surface LAMP-1 has been suggested to be involved in cell migration in breast cancer,^28^ no studies so far showing the correlation of FUT1-modified LAMP-1 and/or LAMP-2 with lysosomal localization and autophagic process. Thus, this is the first report to provide evidence for the involvement of FUT1 in the topographical distribution of LAMP-1 and 2 that subsequently influences the autophagic activity and process of breast cancer cells.

In normal cells, most of the LAMP-1 and 2 are found in the lysosomes and late endosomes that are localized perinuclearly; while a small fraction of the LAMP-1 and 2 is found to shuttle between plasma membrane, endosomes and lysosomes dynamically.^34, 35^ However, in cancer cells, especially those with invasive phenotype, the distribution of lysosomes appears to shift from perinuclear to peripheral pattern for the release of lysosomal contents to facilitate their migration/invasion or metastasis.^36, 37^ In our study, we have found that the majority of LAMP-1 and 2 in FUT1 knockdown breast cancer cells tends to localize at the perinuclear region rather than the cell periphery. Given that LAMPs are required for regulating the motility of lysosomes through dynein-mediated transport along microtubules,^38, 39^ it is plausible that the lack of *α*1,2-fucosylation may alter the integrity of LAMPs, which in turn may affect the dynamic distribution of lysosomes. In addition to lysosomal biogenesis, both LAMP-1 and 2 are also known to participate in autophagic pathway in which knockout of both LAMP-1 and 2 or LAMP-2 alone would result in a massive accumulation of autophagosomes.^15^ However, the enhanced autophagic flux found in our study could not be ascribed to the changes in the expression levels of LAMP-1 and 2 because knockdown of FUT1 failed to significantly alter their protein levels. Taken together, it is more likely that FUT1, which targets LAMP-1 and 2, may exert considerable impact on the positioning of lysosomes as well as the process of autophagy through modification of alpha1,2-fucosylated antigens on these LAMP molecules. However, the possibility cannot be excluded that under-fucosylation of some other target proteins may also participate in these processes.

To date, many factors that participate in the regulation of lysosomal distribution or autophagy have been identified. The preferential accumulation of lysosomes at perinuclear area mainly occurs in the context of starvation and high intracellular pH,^29, 30^ whereas the induction of autophagy generally occurs in response to nutrient deprivation, and metabolic and/or cellular stress.^40, 41, 42^ Recently, Hyung *et al.* has reported that treatment with the inhibitor of *N*-glycosylation, Tunicamycin, resulted in the accumulation of perinuclear lysosomes as well as the induction of autophagy.^43^ This is consistent with the report of Qin *et al.* that Tunicamycin negatively regulates the mTOR signaling pathway and induces autophagy.^44^ However, the specific glycosyltransferases or glycans involved in these cellular processes have yet to be identified in the above-mentioned studies. In this study, the suppression of FUT1, which directs the synthesis of terminal *α*1,2-fucosylation on *N*-glycan backbones, exhibits a very similar phenomenon as observed in Tunicamycin treatment. This implies that *α*1,2-fucosylation alone may be sufficient for modifying lysosomal topography and autophagic process. On the other hand, hypoxic stress is known to be an important upregulator for the induction of autophagy by a HIF-*α*1-dependent manner.^45, 46^ Considering that HIF-*α*1 has been recently reported by Ana *et al.* as an upstream repressor of FUT1 expression in pancreatic tumors,^47^ our identification of the role of FUT1 in autophagy may provide a coherent link between these two findings.

Previously, Jacques *et al.* reported that the presence of *α*1,2-fucosylated antigens (A, B, H, LeB and LeY) appeared to confer tumor growth advantages during the early stage of tumor progression.^48^ Besides, the involvement of *α*1,2-fucosylation in a variety of malignant processes have also been demonstrated. However, only a handful of reports has attempted to elucidate the molecular mechanism involved in these processes. In our study, the finding that *α*1,2-fucosylation contributes to autophagic flux may provide a link to the tumorigenesis, as reduced autophagy is found in tumor cells and is thought to be associated with malignant transformation.^49, 50^ In addition, the role of autophagy in tumor suppression by preventing the accumulation of damaged organelles and macromolecules^49, 51^ further supports our notion that the positive impacts of *α*1,2-fucosylation on tumorigenesis may be partially attributable to its negative effect on autophagy. Although it is known that the *α*1,2-fucosylated LeY antigens can promote cell proliferation and tumorigenesis by activating EGFR/MAPK signaling,^52, 53^ our finding may offer yet another pathway in which *α*1,2-fucosylated LAMP-1 and 2 may regulate autophagy biogenesis, thereby influencing tumor development and progression.

In conclusion, this study has provided the first evidence for a negative impact of FUT1 on autophagic flux in breast cancer cells through its influence on lysosomal positioning. Given the inhibitory effects of autophagy on cell proliferation and tumor development, our work not only provide an alternative mechanism of FUT1-mediated tumorigenesis but also a new perspective on the interplay among *α*1,2-fucosylation, autophagy and tumorigenesis. More importantly, this finding may yield a strong scientific rationale for the future development of FUT1 inhibitor for cancer therapy.

## Materials and Methods

### Reagents and antibodies

Mouse monoclonal antibodies against the human LAMP-1 and 2 were purchased from BD Pharmingen (San Diego, CA, USA; 1 : 2000 dilution) and Santa Cruz Biotechnology (Santa Cruz, CA, USA; 1 : 1000 dilution), respectively. Alexa Fluor 488 mouse anti-human LAMP-1 (for IF staining) was purchased from 53–1079, eBioscience (Vienna, Austria; 1 : 50 dilution). Rabbit polyclonal anti-human LC3 and anti-p62 antibodies were from NBP1–48320 (1 : 1000 dilution) and NBP100–2331 (1 : 500 dilution) Novus Biological (Littleton, CO, USA), respectively. Mouse anti-human LeY IgM was from ab3359, Abcam (Cambridge, UK; 1 : 1500 dilution). Rabbit anti-p70 S6K and mouse anti-phospho-p70 S6K (Thr389) were from #9202 and #9206 Cell Signaling (Danvers, MA, USA; 1 : 1000 dilution), respectively. Control mouse IgG1 antibodies were purchased from R&D Systems (Minneapolis, MN, USA) as an isotype control. AP-conjugated goat anti-mouse IgG and IgM, and streptavidin were obtained from Jackson ImmunoResearch Laboratories (West Grow, PA, USA). Alexa Fluor 488 goat anti-mouse IgG (H +L), Alexa Fluor 594 goat anti-mouse IgG (H +L), Alexa Fluor 594 goat anti-rabbit IgG (H +Land Alexa Fluor 647 goat anti-mouse IgG (H +L) were from Invitrogen (Carlsbad, CA, USA),

### Cell culture and siRNA transfection

Human breast cancer cell line T47D purchased from American Type Culture Collection (ATCC, Manassas, VA, USA) was maintained in RPMI-1640 medium (Gibco, Grand Island, NY, USA) supplemented with 10% FBS and 2 mM Glutamax. MCF-7 (a gift from Michael Hsiao, GRC, Academia Sinica) and MDA-MB-231 cells were grown in Dulbecco's modified Eagle's medium (Gibco) supplemented with 10% FBS and 2 mM Glutamax. For FUT1 siRNA transfection, T47D cells were seeded into six-well plates at a density of 2 × 10^5^ cells per well and cultured overnight. Cells were then transfected with FUT1 siRNA using Lipofectamine RNAiMAX (Invitrogen) according to the manufacturer's instructions. FUT1 siRNA and a scrambled negative control with equivalent GC content (high duplex #2) were synthesized and annealed by Invitrogen. The FUT1 siRNAs used in this work were HSS177655 (sense 5′-AUUAAUGCCCACCCACUCGGGCAGG-3′ and anti-sense 5′-CCUGCCCGAGUGGGUGGGCAUUAAU-3′) and HSS103859 (sense 5′-AAGGCUUAGCCAAUGUCCAGAGUGG-3′ and anti-sense 5′-CCACUCUGGACAUUGGCUAAGCCUU-3′).

### Immunoprecipitation and immunoblotting

Cells were harvested and lysed in lysis buffer (1% Triton X, 20 mM Tris-HCl pH 7.0, 5 mM EDTA, 50 mM NaCl) and 1X complete EDTA-free protease inhibitor mixture (Roche, Basel, Switzerland) followed by centrifugation at 15 000 × *g* for 15 min at 4 °C to remove the cell debri. The supernatants of the cell lysates were then collected and protein concentrations were determined by BCA Protein Assay Kit (Pierce, Rockford, IL, USA). For immunoprecipitation of LAMP-1 and 2, 1 mg of the total lysates were incubated with 2 *μ*g of anti-LAMP-1 or anti-LAMP-2 antibody overnight at 4 °C with gentle rocking followed by 1- h incubation with 50 *μ*l of protein G-Sepharose 4 Fast (GE Healthcare Life Sciences, Buckinghamshire, UK) at 4 °C. The immunoprecipitated samples were then run on 4–12% NuPAGE (Invitrogen) and electrophoretically transferred to a PVDF membrane for immunoblotting. The blots were probed with primary and AP-conjugated secondary antibodies for 1 h each, and immunoreactive bands were visualized using ECF substrate (GE Healthcare Life Sciences). All western blot images were acquired by Typhoon FLA 9500 (GE Healthcare Life Sciences) and intensity of the bands was quantified by ImageQuant 5.2 (GE Healthcare Life Sciences).

### On-membrane click reaction

The respective control vector, FUT1 or FUT2 transfectants were fed with fucose alkyne (kindly provided by Dr Chi-Huey Wong, Academia Sinica, Taiwan) for 3 days. The metabolically labeled cells were harvested, lysed and subjected to immunoprecipitation of the LAMP-1 as described above. The immunoprecipitated LAMP-1 was then separated on 4–12% NuPAGE and transferred to PVDF membranes. The blots were blocked with 3% BSA in PBST buffer for 1 h at RT followed by a click reaction with a mixture of 0.1 mM azido biotin, 0.1 mM Tris-triazoleamine catalyst, 1 mM CuSO4 and 2 mM sodium ascorbate for another 1 h at RT. After this, the membrane was detected by probing with AP-conjugated streptavidin.

### In-gel PNGase F/tryptic digestion

Purified LAMP-1 were run on SDS-PAGE and stained with Coomassie blue R250. The corresponding protein bands were then excised from the gel and degylcosylated by in-gel PNGase F treatment. For in-gel PNGase F digestion, the gel slices were first reduced with 10 mM DTT at 60 °C for 1 h and alkylated with 55 mM iodoacetamide at RT for 1 h followed by soaking with one unit of PNGase F (P0705L, New England Biolabs, Ipswich, MA, USA) in 25 mM ammonium bicarbonate buffer at 37 °C overnight. Afterward, *N*-glycans were extracted two times with pure water and 50% acetonitrile per each. All extracts were pooled together and concentrated by a SpeedVac. The remaining gels were further digested with 12.5 ng/*μ*l trypsin (V5111 Promega, Madison, WI, USA) in 25 mM NH4HCO3 buffer and incubated overnight at 37 °C. The peptide fragments extracted from gels were used for peptide mapping.

### *N-*glycan derivatization and MS analysis

In order to increase MS detection sensitivity with predictable fragmentation pattern and to prevent chromatographic peak splitting because of the anomeric configuration of reducing sugar, the in-gel PNGase F released *N*-glycans were subjected to reduction and permethylation before MALDI-MS profiling and nanoLC-MS/MS analysis. Briefly, the released *N-*glycans were reduced in 50 *μ*l of 1.0 M NaBH_4_/0.05 N NaOH at 45 °C for 2 h. The reaction was quenched by adding drops of glacial acetic acid until no bubbles formation. The reduced *N-*glycan alditols were desalted by passing through Dowex cation exchange resins and recovered from excess of boric acid by co-evaporation with 1 ml of 10% (*v/v*) acetic acid in methanol for three times. Permethylation was carried out by the NaOH/DMSO slurry method at room temperature for 30 min. The permethylated derivatives were recovered by using CHCl_3_/water extraction method. MALDI-TOF MS profiling of the derivatives were carried out in positive-ion mode on a AB Sciex 5800 MALDI-TOF/TOF mass spectrometer (Applied Biosystems, Foster City, CA, USA) using 2, 5-dihydroxybenzonic acid as the MALDI matrix.

NanoLC-MS/MS analyses of the permethylated *N-*glycan alditols were carried out on a homemade nanoLC system consisted of a 50 *μ*m × 4 cm homemade polystyrene-divinylbenzene (PS-DVB) monolithic trap column and a 20 *μ*m × 4 m homemade PS-DVB grafted open tubular analytical column, coupled to Orbitrap Elite (Thermo Scientific, Waltham, MA, USA) MS system. For this nanoLC system, sample was dissolved in 2 *μ*l of 25% (*v/v*) acetonitrile, injected to trap column and then separated in a serially connected analytical column at a constant flow rate of 150 nl/min, with a linear gradient of 10–40% (*v/v*) acetonitrile (with 0.5 *m*M sodium acetate) in 30 min, then increased to 80% acetonitrile in 5 min and held isocratically for another 10 min. The eluent was directly interfaced to a nanospray source based on the liquid junction configuration consisted of an uncoated emitter and a high voltage (in range of 1.5–1.8 kV) platinum electrode. For data-dependent acquisition, the full scan MS spectrum (*m/z* 500–2000) was acquired in the Orbitrap at 60 000 resolution (at *m/z* 400) with automatic gain control (AGC) target value of 5 × 10^6^. Data-dependent acquisition was performed for the 10 most intense ions within the inclusion MS list with intensity threshold of 3000 counts. Subsequently, product-ion dependent CID-MS^3^ experiments were carried out using two distinct B-fragment ion candidates at *m/z* 660.32 and 834.41, with intensity threshold of 100 counts, within 25 most intense ions in MS^2^ spectra. The fragment ion assignment of MS/MS spectra was depicted according to the Domon and Costello nomenclature and interpreted manually.

### Immunofluorescence microscopy

Cells transfected with control and FUT1 siRNA were fixed with 4% paraformaldehyde for 30 min, permeabilized with 0.1% Triton X-100 at room temperature for 15 min and blocked with 1% BSA in PBS for 1 h. The permeabilized cells were incubated with primary antibodies overnight at 4 °C followed by the secondary antibodies for 1 h at room temperature. For double/triple immunofluorescence staining, the sequential staining procedure started with the unlabeled primary antibodies, followed by their corresponding secondary antibodies, and subsequently with the directly conjugated antibodies. Between the sequential staining steps, samples were washed in PBS containing 0.1% Triton X-100 and blocked with 1% BSA. Cell nuclei were visualized by counterstaining with Hoechst 33342. The immunostained samples mounted with Vectashield (Vector Laboratories, Burlingame, CA, USA) were examined under a TCS SP8 confocal microscopy (Leica, Wetzlar, Germany). Fluorescence images were acquired with LAS AF Software (Leica).

### Quantification of lysosomal distribution

As previously described,^30^ the intracellular distribution of lysosomes can be divided into two main groups that are defined as peripheral- and perinuclear-dominant lysosomal distribution if >50% of LAMP-1(+) vesicles localized in peripheral and perinuclear region, respectively. To evaluate the proportion of cells with these two distribution patterns, cells stained for LAMP-1 in 7–10 random fields were classified and counted. Note that LAMP-1-positive vesicles were referred to as lysosomes and the data were expressed as the percentage of cells with peripheral-dominant lysosomal localization and normalized to control group. Quantification was carried out on approximately 80–100 cells per sample group from two to three independent experiments.

### Quantification of LC3 and LAMP-1 colocalization

Cells transfected with control and FUT1 siRNA were co-immunostained for endogenous LAMP-1 and LC3 as described above in immunofluorescence microscopy assay. Seven to 10 fields of each sample were selected randomly and captured under a TCS SP8 confocal microscopy. To analyze the colocalization between LC3 and LAMP-1, a constant threshold between two channels of confocal images was applied and the integrated pixel intensity of colocalization regions was quantified using the Measure Colocalization in MetaMorph software (version 7.8.4.0) (Molecular Devices, Sunnyvale, CA, USA). All values were expressed as the percentage of LC3/LAMP-1 colocalization to the total number of LC3 puncta, and subsequently normalized to the control for comparison. Quantification was carried out on approximately 30 cells per sample group from three independent experiments.

## Abbreviations

FUT1: *α*1,2-fucosyltransferases
LAMP-1 or 2: lysosomal-associated membrane protein 1 or 2
LeY: blood group Lewis Y antigen
LC3: microtubule-associated protein 1A/1B-light chain 3
p62: nucleoporin p62 (also called SQSTM1, sequestosome 1)
mTORC1: mammalian target of rapamycin complex 1
