# Supplementary material for: Fucosylation of LAMP-1 and LAMP-2 by FUT1 correlates with lysosomal positioning and autophagic flux of breast cancer cells
Source: Cell Death Dis. 2016 Aug 25;7(8):e2347–. doi: 10.1038/cddis.2016.243 (PMC5108328; doi:10.1038/cddis.2016.243)
Supplement: Supplementary Information [file cddis2016243x1.docx]

**Supplementary Figure S1**

**
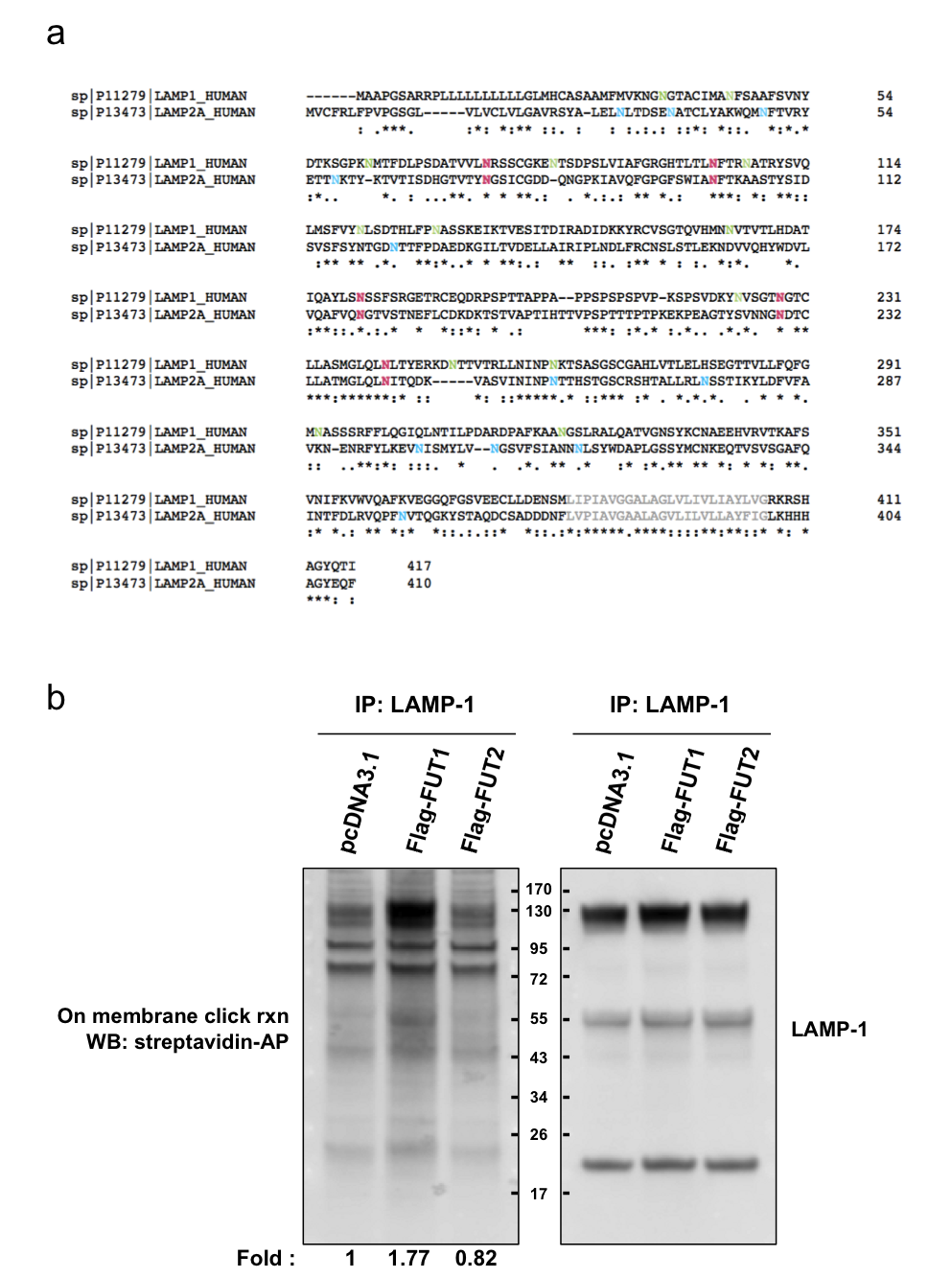
**

**Supplementary Figure S2**


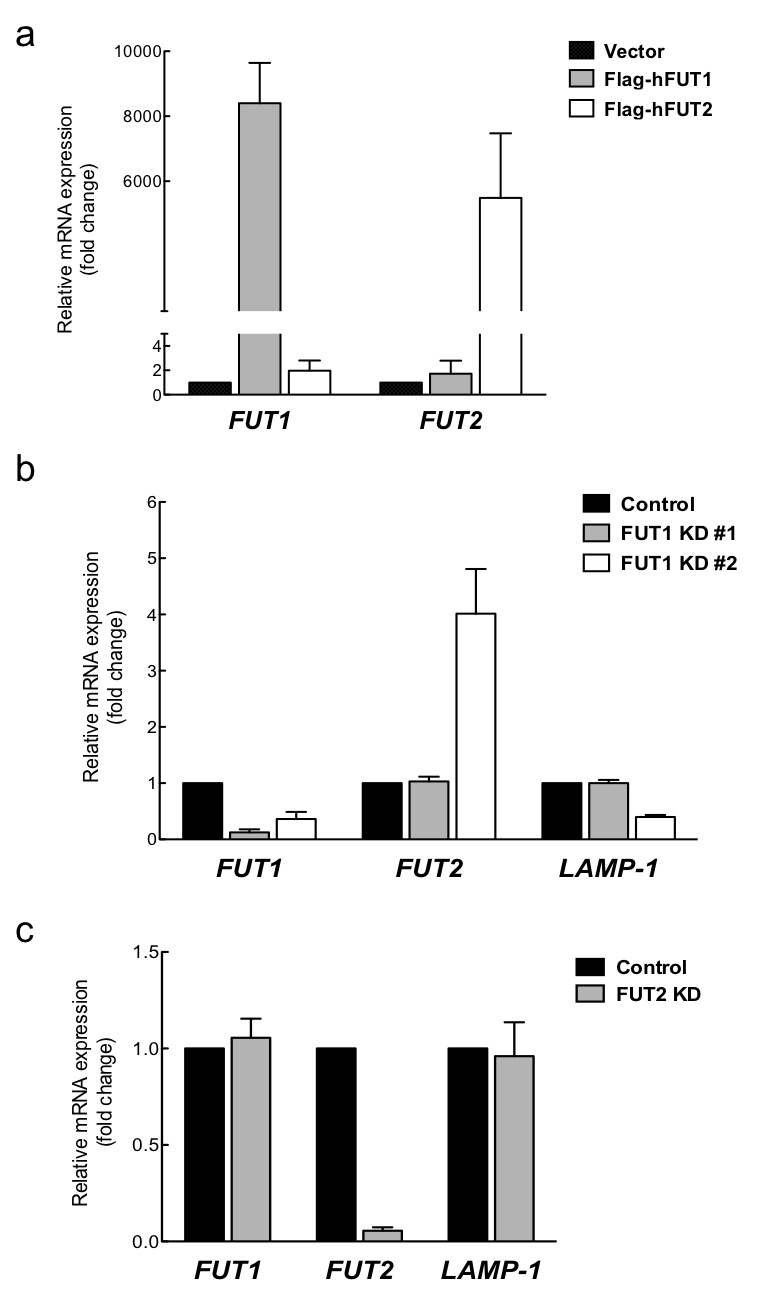


**Supplementary Figure S3**

**
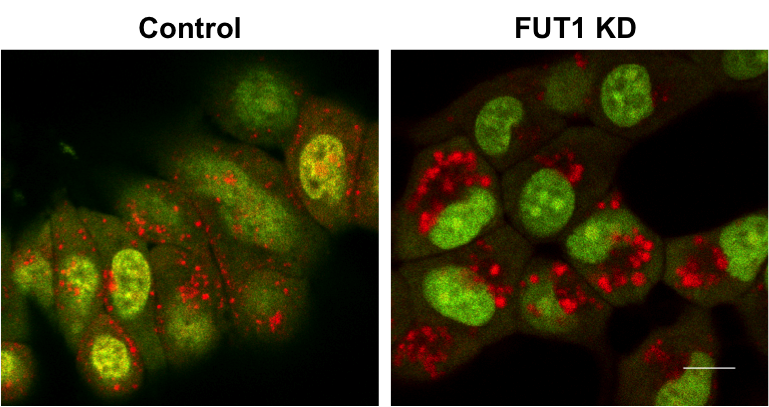
**

**Supplementary Figure S4**

**
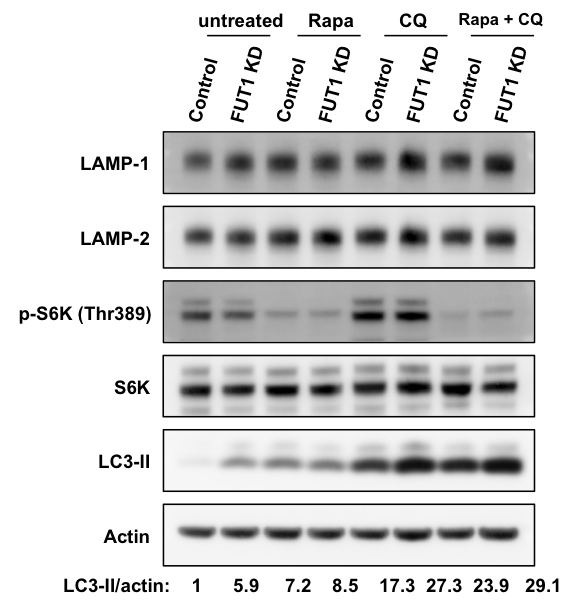
**

**Supplementary Figure S5**

**
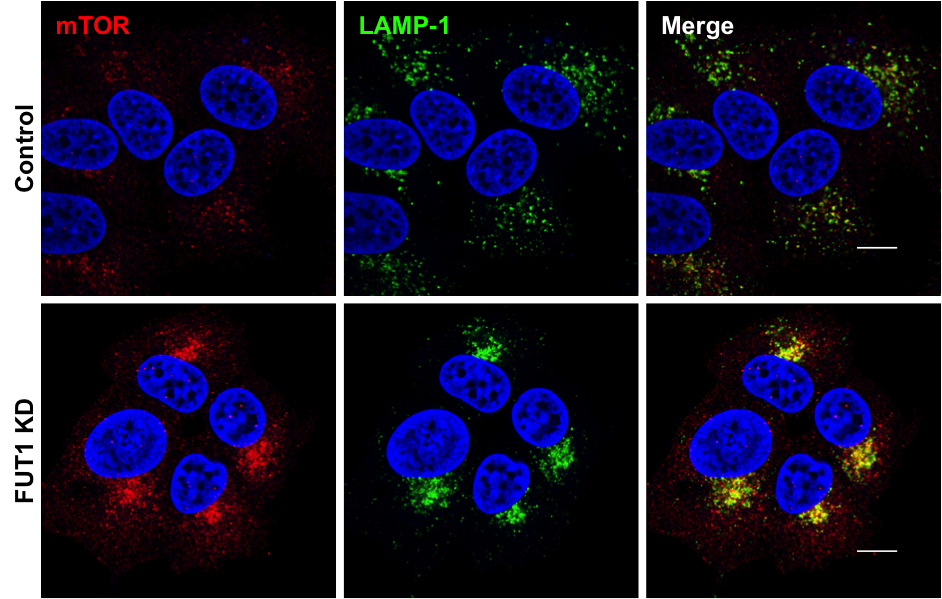
**

**Supplementary Table S1**

| Table 1. Summary of analytical methods used to measure the fucosylation levels of immunoprecipitated LAMP-1 and LAMP-2  from the control and FUT1 knockdown T47D cells | | | |
| --- | --- | --- | --- |
|  | Methods | Information available | Results |
| LAMP-1 | Western blot with anti-LeY  MALDI-TOF profiling of mock sample  Targeting LC-MS^3^ analysis of bi-antennary glycan | Level of LeY  Full MS (m/z 1400 - 4000)  1) MS^3^_­_ spectra of H2, LeX and  LeY antigens  2) Quantitative extracted ion  chromatograms (EICs) | 🡻 Expression of LeY upon FUT1 KD  Highly fucosylated bi-, tri- and tetra-antennary N-glycans along with a comparatively low level of sialylation was found on mock-treated LAMP-1  🡻 H2 and LeY levels of bi-antennary N-glycans  upon FUT1 KD |
| LAMP-2 | Western blot with anti-LeY    MALDI-TOF profiling | Level of LeY  Full MS (m/z 2200 - 4000) | 🡻 Expression of LeY upon FUT1 KD  🡻 Fucosylation level of tri- and tetra-antennary  N-glycans upon FUT1 KD |

**Supplementary Figure Legends**

**Figure S1. Detection of fucosylation on LAMP-1 by On-membrane click reaction method.** (a) Schematic representation of potential N-glycosylation sites on LAMP-1 and LAMP-2. The putative N-glycosylation sites that are conserved between LAMP-1 and LAMP-2 are highlighted in red, and their individual N-linked sites are highlighted in green and blue, respectively. NetNGlyc 1.0 server was used to identify the prediction sites. (The amino acid sequences of LAMP-1 and LAMP-2 were retrieved from the UniProtKB database and were aligned by Clustal Omega program. Transmembrane domains are shaded in gray). (b) Overexpression of FUT1 but not FUT2 enhanced fucosylation of LAMP-1. T47D cells over-expressing FUT1 or FUT2 were incubated with alkynyl fucose for 3 days, and cells were lysed. LAMP-1 was immunoprecipitated from the cell lysates and subjected to SDS-PAGE followed by On-membrane click reaction with azido biotin for the subsequent immunoblotting with AP-conjugated streptavidin. The same blots were stripped and reprobed for LAMP-1. Fold changes relative to control were calculated by using the ratio of biotin to immunoprecipitated LAMP-1.

**Figure S2. mRNA expression of *FUT1*, *FUT2* and *LAMP-1 in overexpressing or silencing T47D cells.*** (a) Real-time RT-PCR analysis of FUT1 and FUT2 in T47D cell transfected with pcDNA3.1 (control vector), pcDNA3.1-Flag-FUT1 or pcDNA3.1-Flag-FUT2. The mRNA levels were normalized to GAPDH mRNA and compared to control cells. (b) mRNA expression levels of FUT1, FUT2 and LAMP-1 in T47D cells transfected with control scrambled or FUT1-specific siRNAs (siFUT1#1 and siFUT1#2). The expression levels were compared to control siRNA-treated cells after normalization with HPRT1 mRNA. (c) mRNA levels of FUT1, FUT2 and LAMP-1 in FUT2 knockdown T47D cells. All data were expressed as fold-change of expression levels of control cells from three independent experiments. Bars represent the means ± SD.

**Figure S3. FUT1 knockdown leads to an increase in size of acidic compartments.** Control and FUT1 knockdown T47D cells were stained with acridine orange (AO, 5μg/ml) for 30 min at 37°C. The cells were then excited by blue light for red (AO in acidic endosomal/ lysosomal compartments) and green (AO in cytosol and nuclei) fluorescence detection. Magnification: 63X (zoom 1.5X). Scale bars, 10µm.

**Figure S4. Measurement of LC3 levels under autophagy induction and/or inhibition.** LC3-II levels and mTOR activity of control and FUT1 knockdown MCF-7 cells were evaluated after treatment with chloroquine (CQ), rapamycin (Rapa) or both for 16h. Whole cell lysates were then collected and subjected to immunoblot analysis with antibodies against LAMP-1, LAMP-2, phospho-p70 S6K (Thr389), total S6K and LC3II. Actin was used as a loading control. Autophagy activity was determined by densitometry analysis of LC3-II levels relative to actin.

**Figure S5. FUT1 knockdown facilitates the perinuclear accumulation of mTOR.** Control and FUT1 knockdown MCF-7 cells were co-stained with anti-LAMP-1 (green) and anti-mTOR (red), followed by staining the nuclei with Hoechst (blue). Stained cells were then analyzed by confocal microscopy at 63X magnification (zoom 2.5X). Scale bars, 10µm.
